# Risk factors of transmission and natural history of chronic hepatitis B infection in Iranian patients 

**Published:** 2019

**Authors:** Afsaneh Sharifian, Sara Ashtari, Behzad Hatami, Seyed Reza Mohebbi, Nosratollah Naderi

**Affiliations:** 1 *Basic and Molecular Epidemiology of Gastrointestinal Disorders Research Center, Research Institute for Gastroenterology and Liver Diseases, Shahid Beheshti University of Medical Sciences, Tehran, Iran*; 2 *Gastroenterology and Liver Diseases Research Center, Research Institute for Gastroenterology and Liver Diseases, Shahid Beheshti University of Medical Sciences, Tehran, Iran*; 3 *Foodborne and Waterborne Diseases Research Center, Research Institute for Gastroenterology and Liver diseases, Shahid Beheshti University of Medical Sciences, Tehran, Iran*

**Keywords:** Chronic hepatitis B, Risk factors, Natural history, Iran

## Abstract

**Aim:**

To investigate routes of transmission, demographic characteristics, and frequency of different phases of chronic hepatitis B (CHB) in 2000 Iranian patients.

**Background:**

Knowledge about the most frequent risk factors of CHB and its different phases is very important for optimal prevention and management policy making.

**Methods:**

In this cross-sectional study, 2000 HBsAg positive patients who were referred to Taleghani Hospital from 2011 through 2018 were enrolled. ELISA method was employed to detect serological markers of CHB. Taking into account the HBV DNA and ALT levels and HBeAg status, the patients were classified in four groups, according to AASLD 2017 guideline.

**Results:**

Male and female patients had nearly equal frequencies in our study and 82.5 % of them aged more than 20 years. A great number of our patients (95%) were HBeAg negative and the most frequent risk factors of HBV infection were positive periodontal and family history (40.3% and 24.9%, respectively). The majority of our patients were inactive carriers (63.35%), while s mall number of them were in the immune tolerant group (2.15 %).

**Conclusion:**

Immune tolerance phase group had the minimum number of members in our study and most of them were above 20 years old. This can be due to the mass vaccination of neonates since 1993. Most of CHB patients were in inactive carrier group. Although it is recommended not to treat these patients, performing periodic liver function tests and disease severity assessment is warranted, especially in patients above 40 years old**.**

## Introduction

 Chronic hepatitis B (CHB) is defined as HBsAg seropositivity for 6 months or beyond. CHB has a dynamic and complicated course (1). According to the World Health Organization (WHO) report, in spite of available vaccination against hepatitis B virus (HBV), about 257 million people are infected with HBV and 887,000 patients die annually due to HBV complications, cirrhosis and hepatocellular carcinoma (HCC) (2). The global prevalence of HBV varies from less than 2% to more than 8% in different parts of the world. In Iran, the prevalence of CHB ranges from 0.8%-1.3% among children and adolescents (3) to 2%-4% or even more in older patients (4-6). Overall, near two million Iranian adults are chronically infected with HBV and we have to keep in mind that average age of infected individuals has increased (7, 8). 

Complex interaction between viral, host, and environmental factors affects the natural history of CHB which may lead to cirrhosis and HCC (8-11). Taking into account the hepatitis B e antigen (HBeAg) status, HBV DNA level (viral load) and alanine aminotransferase (ALT) level, chronic HBV infection can be classified into different clinical phases.

HBV is transmitted by perinatal, percutaneous, and sexual exposure. Major routes of HBV transmission in high risk areas are perinatal and percutaneous, while in intermediate and low risk countries percutaneous and sexual exposure are the main risk factors. In hyper endemic areas, especially among children, close person-to-person contacts (presumably by open cuts and sores) is a significant way of transmission (9). Knowledge about the frequency of different phases of HBV infection is important for assessing financial, human resources, and equipment requirements for better management planning of CHB. Meanwhile, it is critical to determine the current predominant routes of HBV transmission to prevent its spread. This study was designed to evaluate these parameters. 

## Methods

**Patient population**

Since Tehran’s Taleghani Hospital is one of the main referral centers for liver diseases in Iran, 2000 CHB patients from different parts of Iran were referred to it from 2011 to 2018. They were enrolled in this cross-sectional study, based on HBsAg positivity over at least six months. All participants gave their written informed consent to be included in the study. The patients’ demographic information and medical records, including name, age, sex, marital status, family history of CHB, cirrhosis or HCC, education, occupation, ethnicity, routes of transmission and laboratory tests were collected via a questionnaire filled by a trained researcher. This study was approved by the Ethics Committee of Research Institute for Gastroenterology and liver Diseases, Shahid Beheshti University of Medical Sciences. 

**Laboratory methods**

Four ml blood sample was obtained from each participant and added into EDTA tube to be sent to laboratory for serum separation. ELISA and Phenol-chloroform extraction methods were obtained to determine Alanine aminotransferase (ALT) level and extract DNA from blood samples. Before performing HBV DNA quantitative PCR, the extracted DNA was stored at -20°C. PCR and semi-nested PCR were used to amplify HBV DNA and HBV cDNA respectively. Afterward, the fragments were detected on 1% w/v agarose gel. The gels were investigated by UV transillumination or white light. If samples had detectable HBV DNA, quantitative real-time PCR was used to determine the quantitative levels of HBV DNA. HCV and HDV statuses were determined via HCV and HDV Ab assessment (ELISA methods). At the end, patient phases of HBV infection were determined, using AASLD 2017 guideline (12).

**Statistical methods**

Categorical variables were expressed as numbers and percentages. Chisquare or Fisher’s exact test, where appropriate, was used for analyzing categorical variables. Continuous variables were expressed as medians or means, standard deviation (SD), and 95% confidence interval (CI) as appropriate. All analyses were performed using SPSS version 21.0 (SPSS INC, Chicago, IL, USA). A twotailed P < 0.05 was considered as statistically significant. 

## Results

A total of 2000 CHB patients were included in this study from whom 1056 (52.8%) were male and 944 (47.2%) were female. The mean age of the patients was 43.9 ± 15.2 (SD) years old (ranging from 5 to 84). The majority of patients, 1900 (95.0%) were HBeAg negative. Demographic characteristics and routes of CHB transmission are shown in Tables 1-3. Most of the HBeAg positive patients were 20-60 years old, and none of them was older than 60 years. Co-infection with hepatitis C and D virus was seen in 39 (1.9 %) and 84 (4.2%) patients, respectively. HBeAg negative patients were generally older than HBeAg positive patients (P<0.01). In the present study, the most frequent risk factors for HBV infection were positive periodontal and family histories (40.3% and 24.9%, respectively), followed by a history of cupping (9.2 %). These factors had statistically significant differences between HBeAg negative patients and HBeAg positive patients (P<0.05). No significant differences were detected in sex, history of vaccination, marriage status, education, occupation and other risk factors between HBeAg negative patients and HBeAg positive patients (P <0.05). The frequency of CHB phases in the study population and their distribution based on age and sex groups are listed in Table 4.

**Table 1:** Demographic characteristics of the study population (n=2000)

Demographic characteristics		HBeAg-Positive (n=100)	HBeAg-Negative (n=1900)	*P*-value
Gender	Male Female	60 (60) 40 (40)	996 (52.4) 904 (47.6)	0.139
Age (years)	<20 20-40 40-60 >60	9 (9) 45 (45) 46 (46) 0 (0)	60 (3.2) 747 (39.3) 812 (42.7) 281 (14.8)	<0.001*
History of Vaccination	Yes No	16 (16) 84 (84)	290 (15.3) 1610 (84.7)	0.84
Anti-HCV (+)		3 (3)	36 (1.9)	0.43
Anit-HDV (+)		6 (6)	78 (4.1)	0.35
Marriage	Single Married Divorced	19 (19) 80 (80) 1 (1)	280 (14.7) 1593 (83.8) 27 (1.4)	0.48
Education	Illiterate Under Diploma Diploma Bachelor & Upper	19 (19) 30 (30) 27 (27) 24 (24)	355 (18.7) 599 (31.5) 609 (32.1) 337 (17.7)	0.40
Ethnicity	Fars Kurd Lor Turk Baloch Torkaman Arab Gilani Mazandaran Afghan Bako	36 (36) 10 (10) 9 (9) 17 (17) 0 (0) 1 (1) 0 (0) 1 (1) 7 (7) 17 (17) 2 (2)	662 (34.8) 81 (4.3) 164 (8.6) 584 (30.7) 25 (1.3) 7 (0.4) 7 (0.4) 33 (1.7) 89 (4.7) 240 (12.6) 8 (0.4)	0.01*
Occupation	Worker Employee Self-employment Jobs with high risk^≠^ Housewife Retired Student Unemployed	15 (15) 13 (13) 21 (21) 1 (1) 30 (30) 9 (9) 11 (11) 0 (0)	202 (10.6) 197 (10.4) 416 (21.9) 138 (7.3) 680 (35.8) 142 (7.5) 98 (5.2) 27 (1.4)	0.23

*(P < 0.05), ^≠^ health care workers such as surgeons, nurses and dentists, policemen, barbers and drivers.

**Table 2:** Risk factors for chronic hepatitis B virus infection in the study population (n=2000)

Risk Factors	HBsAg-Positive	HBeAg-Positive	HBeAg Negative	*P*-value
Hemodialysis	4 (0.1)	0 (0)	4 (0.1)	0.64
Sexuality	17 (0.4)	1 (0.5)	16 (0.4)	0.86
Blood slashing	56 (1.8)	0 (0)	56 (1.5)	0.08
Needle stick	80 (2.1)	1 (0.5)	79 (2.2)	0.11
Transfusion	107 (2.8)	2 (1.1)	105 (2.9)	0.12
Tattooing	167 (4.4)	4 (2.2)	163 (4.5)	0.10
IV drug abuse	179(4.7)	10 (5.5)	169 (4.7)	0.70
Shaving	330 (8.7)	12 (6.7)	318 (8.8)	0.21
Cupping	351 (9.2)	13 (7.3)	338 (9.4)	0.22
Family History	948 (24.9)	70 (38.9)	878 (24.4)	<0.001*
Periodontal	1534 (40.3)	67 (37.3)	1467 (40.8)	0.01*
Total	3809 (100)	180 (100)	3593 (100)	7582 (100)

*(P < 0.05)

## Discussion

HBV is one of the most serious infectious diseases in the world whose prevention and management remains a global health problem (10). In our study, there was no significant difference in the frequency between male and female patients (P<0.05).

 This was congruent to a study by Farhat et al. (11), but in contrast to another study by Poorolajal et al. (3) who found a significant difference between sex groups. Most of our patients were at 40-60 (42.9%) age group followed by 20-40 (39.65%) age group. Other reviews have also shown that prevalence of chronic HBV infection is higher in middle aged and elders, as compared to children, teenagers, and youth (3).

**Table 3:** Overlap of risk factors for chronic hepatitis B virus infection in the study population (n=2000)

Risk factors	Frequency	Percent	Valid Percent
Cupping +Tattooing	4	.2	.2
Cupping + Periodontal	101	5.1	5.1
Cupping + Transfusion	9	.5	.5
Cupping + Family History	8	.4	.4
Tattooing + Periodontal	67	3.4	3.4
Tattooing + Sexual	16	.8	.8
Tattooing + Transfusion	4	.2	.2
Tattooing + Family History	9	.5	.5
Needle stick + Periodontal	12	.6	.6
Needle stick + Shaving	6	.3	.3
Needle stick + Blood splashing	6	.3	.3
Periodontal + Shaving	59	3.0	3.0
Periodontal + Blood splashing	23	1.2	1.2
Periodontal + Transfusion	14	.7	.7
Periodontal + IV drug abuse	22	1.1	1.1
Periodontal + Family History	552	27.6	27.6
Sexual + Blood splashing	8	.4	.4
Sexual + Family History	7	.4	.4
Shaving + IV drug abuse	4	.2	.2
Shaving + Family History	79	4.0	4.0
Blood splashing + Transfusion	7	.4	.4
Transfusion + Family History	6	.3	.3
IV drug abuse + Family History	21	1.1	1.1
Cupping + Periodontal + Family History	27	1.4	1.4
Tattooing + Periodontal + Transfusion	9	.5	.5
Not find	920	46.0	46.0
TOTAL	2000	100.0	100.0

**Table 4:** Phases of chronic HBV infection based on age groups and gender

Phases of CHB	Number (%)		Male	Female	Age groups			
					< 20	20-40	40-60	>60
HBeAg Positive								
Immune tolerance	43 (2.15%)		29	14	8	16	19	0
Immune active	57 (2.85%)		31	26	1	30	26	0
HBeAg Negative								
Inactive carrier	1267 (63.35%)		712	556	41	515	552	160
Immune active	633 (31.65%)		285	348	19	232	261	121

The very important and alarming point in this study was that 84% of these patients had a negative history for vaccination, which is consistent with our previous studies (13). This suggests that we have to enforce vaccination programs, both in high and low risk groups. Co-infection with HDV or HCV is associated with reduced survival and increased risk of progression to liver complications in CHB patients. In our study, anti-HDV and anti-HCV were positive in 84 (4.2%) and 39 (2%) of patients. These rates are similar to other Iranian studies (14, 15). Lower rates of HDV or HCV co-infection in Iran compared to other countries (Pakistan12.7%, Turkey8.5% and United states 9.3%) may be due to our lower detection rate or the higher rate of vaccination as well as social and cultural development (2,6). 

Although the majority of dentists have relatively high level of knowledge about HBV transmission routes (16), the most frequent risk factor in our study (40.3%) was positive periodontal history. Although this may be an incidental finding, it still calls for better education and supervision in this area. The second most frequent risk factor in our study was positive family history (24.9%). This is in concordance with other studies in which family history was the most frequent route (17, 18). This indicates that we have to enforce our screening and vaccination programs for pregnant women and family members of HBV patients, and insist on patients’ education to prevent intra-familial spread. In a case control study of 500 CHB subjects, they found that certain jobs (police, barbers, and drivers) were independent risk factors for HBV infection (19), but we did not find such a correlation in our study. On the other hand, higher rates of CHB among 20-60-year-old group apart from inadequate vaccination rates may be related to high-risk behaviors such as having multiple sexual partners, tattooing, cupping, and IV drug abuse (19). Thus, apart from the national vaccination programs, focus should be directed towards elimination of such high-risk behaviors. Also, we have to bear in mind that a considerable number of patients have more than two risk factors for CHB (17). In our study, more than half of the patients had more than two2 risk factors as shown in Table 3. 

In the current study, the frequency of HBeAg negative CHB was significantly higher than that of HBeAg positive CHB (95% vs. 5%), which is significantly lower than those reported in previous years in Iran (13, 20, 21). Meanwhile, this is completely in agreement with recent studies in Europe, Asia, and the United States which observed an increase in HBeAg negative and reduction in HBeAg positive CHB prevalence (22-26). This shift may be related to the successful vaccination programs which lead to a decline in new HBV infection cases, improved detection and effective treatment strategies as well as increase in mutations in the pre C region of the virus (27-29). Our results showed that HBeAg negative patients were generally older than HBeAg positive cases (P<0.01) which was fully consistent with other studies (21, 30, 31). 

Chronic HBV infection can be classified into 4 different clinical phases, according to AASLD 2017 guideline; Immune tolerance, HBeAg positive Immune active, Inactive carrier, and HBeAg negative Immune active (12). Note that these phases may not occur in all patients or sequentially. In addition, the time spent in different phases differs and transition from one phase to the next may be so fast that one may not recognize them in clinical practice. 

The majority of our patients were inactive carriers (63.35%). This phase may last for the lifetime without reactivation of HBV infection or HBsAg seroconversion. Although based on recent guidelines it is not recommended to treat these patients, periodic liver function tests and disease severity assessment should be considered especially in patients above 40 years old (32, 33). The second most frequent phase in this study was Immune active which requires either intense workup or treatment. The minority of our patients were in the immune tolerance phase (2.15%). This may indicate successful vaccination of neonates in our country. In our study, 44% of immune tolerant patients were older than 40 years. Immune tolerant patients usually remain in this phase for 1 to 4 decades, but increasing age has been shown to predict adverse outcomes in this group. Hence liver biopsy and monitoring HBeAg and liver function tests, every 3-6 months, has been suggested for immune tolerant patients who are older than 40 years (34, 35). 

To the best of our knowledge, this study was the largest of its kind in Iran; however, some limitations exist. As the survey were cross-sectional, it was not possible to collect data on transitions between different phases of CHB; therefore, designing a cohort study for future is suggested. 

In summary, positive periodontal and family histories were cardinal risk factors for CHB in our study. This emphasizes that although neonatal vaccination since 1993 has decreased the prevalence of HBV in younger population (2), there is still room for improving vaccination programs and educating medical personnel about preventive methods as well as patients about vertical and intra-familial spread. The vast majorities (95%) of CHB patients in our study were HBeAg negative and most of them were inactive carriers. Although they seldom need treatment, periodic monitoring of liver function tests and disease severity assessment, especially in patients above 40 years old, is warranted. In addition, positive periodontal and family histories were the most frequent risk factors in this study demonstrating the need for more supervision and effort in these areas.
